# Plasma CD16^+^ Extracellular Vesicles Associate with Carotid Artery Intima-Media Thickness in HIV^+^ Adults on Combination Antiretroviral Therapy

**DOI:** 10.1128/mbio.03005-21

**Published:** 2022-04-18

**Authors:** Erika G. Marques de Menezes, Xutao Deng, Jocelyn Liu, Scott A. Bowler, Cecilia M. Shikuma, Mars Stone, Peter W. Hunt, Lishomwa C. Ndhlovu, Philip J. Norris

**Affiliations:** a Vitalant Research Institute, San Francisco, California, USA; b Department of Laboratory Medicine, University of California, San Francisco, California, USA; c Division of Infectious Diseases, Department of Medicine, Weill Cornell Medicine, New York, New York, USA; d Department of Tropical Medicine, University of Hawaii, Honolulu, Hawaii, USA; e Department of Medicine, Hawaii Center for AIDS, John A. Burns School of Medicine, University of Hawaii, Honolulu, Hawaii, USA; f Department of Medicine, UCSF, San Francisco, California, USA; Johns Hopkins Bloomberg School of Public Health

**Keywords:** apoptosis, cardiovascular disease, carotid intima-media, endothelial cells, extracellular vesicles, human immunodeficiency virus, monocytes

## Abstract

HIV-infected individuals have increased risk for cardiovascular disease (CVD) despite suppressive antiretroviral therapy (ART). This is likely a result of persistent immune activation and systemic inflammation. Extracellular vesicles (EVs) have emerged as critical mediators of intercellular communication and may drive inflammation contributing to CVD. EVs were characterized in plasma from 74 HIV-infected individuals on combination antiretroviral therapy (cART) and 64 HIV-uninfected controls with paired carotid intima-media thickness (cIMT) assessment. EVs were profiled with markers reflecting lymphoid, myeloid, and endothelial origin. Seventeen plasma inflammatory biomarkers were also assessed. Human umbilical vein endothelial cell (HUVEC) apoptosis was quantified after EV exposure. A significant correlation was observed in HIV-infected participants between cIMT and EVs expressing CD16, and the monocyte-related markers CD4, CD14, and CX3CR1 showed a similar but nonsignificant association with cIMT. No significant correlation between cIMT measurements from HIV-uninfected individuals and EVs was observed. Levels of serum amyloid A, C-reactive protein, and myeloperoxidase significantly correlated with CD14^+^, CD16^+^, and CX3CR1^+^ EVs. No correlation was noted between cIMT and soluble inflammatory markers. HUVECs showed increased necrosis after exposure to the EV-containing fraction of plasma derived from HIV-infected individuals compared to uninfected controls. Our study reveals that EVs expressing monocyte markers correlated with cIMT in HIV-infected individuals on cART. Moreover, EV fractions derived from HIV-infected individuals lead to greater endothelial cell death via necrotic pathways. Collectively, EVs have potential as biomarkers of and therapeutic targets in the pathogenesis of CVD in the setting of treated HIV disease.

## INTRODUCTION

HIV-infected individuals have a 2-fold-increased risk of cardiovascular disease (CVD) compared with the general population (1, 2). Potentially contributing factors include HIV-related risk factors, such as the direct effect of HIV, the proinflammatory impact of residual virus despite combination antiretroviral therapy (cART), potential adverse effects of antiretroviral therapy (ART), and inflammation, and traditional risk factors, such as diabetes mellitus and smoking (3, 4). These CVD risk factors are hypothesized to lead to atherosclerosis via mechanisms including T cell activation and/or senescence (5), endothelial activation (6), and coagulation abnormalities and platelet activation (7, 8).

Monocytes play an essential role in HIV disease and atherogenesis (9, 10). Migration of monocytes into early atherogenic lesions is a key feature of the development of atherosclerosis, a process that may be accelerated by HIV infection (11). Soluble vascular cell adhesion molecule (sVCAM) (10) and expansion of transitional monocytes are associated with carotid intima-media thickness (cIMT), a direct measure of atherosclerosis, independent of traditional CVD risk factors (10–12). The SUN study found that an increase in circulating intermediate and nonclassical monocytes predicted greater coronary artery calcification progression, independent of traditional and HIV-related risk factors (13). However, the majority of people living with HIV on stable cART are still at increased risk for CVD, although the underlying immunologic mechanisms and effective biomarkers of this process remain unresolved.

Extracellular vesicles (EVs) have emerged as important mediators in cell-cell communication (14, 15). EVs are derived from parent cells and represent a heterogeneous population, including microvesicles that bud from the cell membrane and exosomes that have endosomal/intracellular organelle origin (16). EVs are angiogenic, procoagulant, and pro- or anti-inflammatory (17). Studies have demonstrated that monocyte-derived EVs induce apoptosis in endothelial cells and promote coagulation pathways by tissue factor release (18). Monocyte-derived EVs can also promote endothelial cell migration by miR-150 delivery (19), although EVs from macrophages have also been reported to suppress integrin-mediated endothelial cell migration (20). Macrophage-derived EVs may also promote leukocyte rolling and adherence via elevation of intercellular adhesion molecule (ICAM-1) and NF-κB activation (21). EVs derived from endothelial cells, platelets, and monocytes have been shown to be elevated in HIV infection and to activate human umbilical cord vein endothelial cells (HUVECs) *in vitro* (22). However, limited data exist about EVs as predictors of atherosclerosis in HIV-infected subjects at risk for CVD.

Most soluble biomarkers do not contain information about their cellular origin, but EVs can be phenotyped using surface markers specific for the parent cell type. The specific cellular origin of the EV can reflect cellular functions and reveals a specific signature of cellular activation and injury (23, 24). However, the role of EVs as biomarkers for CVD or their role in the pathogenesis of CVD in the setting of treated HIV disease is not fully characterized. This study assessed the relationship between cIMT and EV phenotype and soluble inflammatory biomarkers in HIV-infected individuals on cART and determined EV effect on endothelial cell activation and death.

## RESULTS

### Demographic and clinical characteristics.

Seventy-four cART-suppressed HIV-infected individuals and 64 HIV-uninfected controls were examined in this study (Table 1). The majority of the participants were male, with a mean body mass index (BMI) of 27 kg/m^2^. The mean age was slightly older in uninfected controls than in ART-suppressed participants (54 versus 50 years, *P* = 0.01). HIV-infected participants had a mean CD4 T cell count of 516 cells/μL, and only 8 had detectable plasma HIV RNA (range, 53 to 132 copies/mL). All participants had common carotid artery (CCA)-IMT and bifurcation (BIF)-IMT values that were similar to and lower than 0.9 mm, indicating normal IMT values. The mean predicted Framingham risk scores (FRS) of the HIV-infected individuals and uninfected controls were 0.08 ± 0.06 and 0.05 ± 0.04, respectively. The majority of study participants had a low FRS (25), and the HIV-infected participants were more likely to be taking a statin drug (33% versus 12%, *P* = 0.02).

**TABLE 1:** Characteristics of the study population^*a*^

Participant characteristic	Uninfected (*n* = 64)	HIV infected (*n* = 74)	*P* value
Demographics			
Male [*n* (%)]	52 (81)	68 (92)	NS
Age (yr) (mean ± SD)	54 ± 8	50 ± 7	0.01
BMI (kg/m^2^) (mean ± SD)	27 ± 5	27 ± 5	NS
Framingham risk score (ATP 3) (mean ± SD)	0.05 ± 0.04	0.08 ± 0.06	0.006
Diabetes mellitus [*n* (%)]	3 (4.7)	8 (11)	NS
Smoking history [*n* (%)]	39 (61)	53 (83)	NS
Statin use [*n* (%)]	8 (12)	21 (33)	0.02
Viral-immunological			
HIV viral load detectable [*n* (%)]	NA	8 (11)	
CD4^+^ T cell count (cells/μL) (mean ± SD)	ND	516 ± 283	
Nadir CD4^+^ T cell count (cells/μL) (mean ± SD)	NA	181 ± 180	
CD8^+^ T cell count (cells/μL) (mean ± SD)	ND	759 ± 323	
History of AIDS [*n* (%)]	NA	24 (32)	
Cardiac imaging [mean (interquartile range)]			
Right common carotid (mm)	0.8 (0.7–0.9)	0.7 (0.7–0.8)	NS
Right bifurcation (mm)	0.8 (0.6–0.9)	0.8 (0.6–0.9)	NS

a *P* values were determined by the Mann-Whitney test. Abbreviations: NS, nonsignificant (*P* > 0.05); BMI, body mass index; ATP 3, adult treatment panel 3; NA, not available; ND, not done.

### Association between plasma EV phenotype and cIMT.

**FIG 1:**
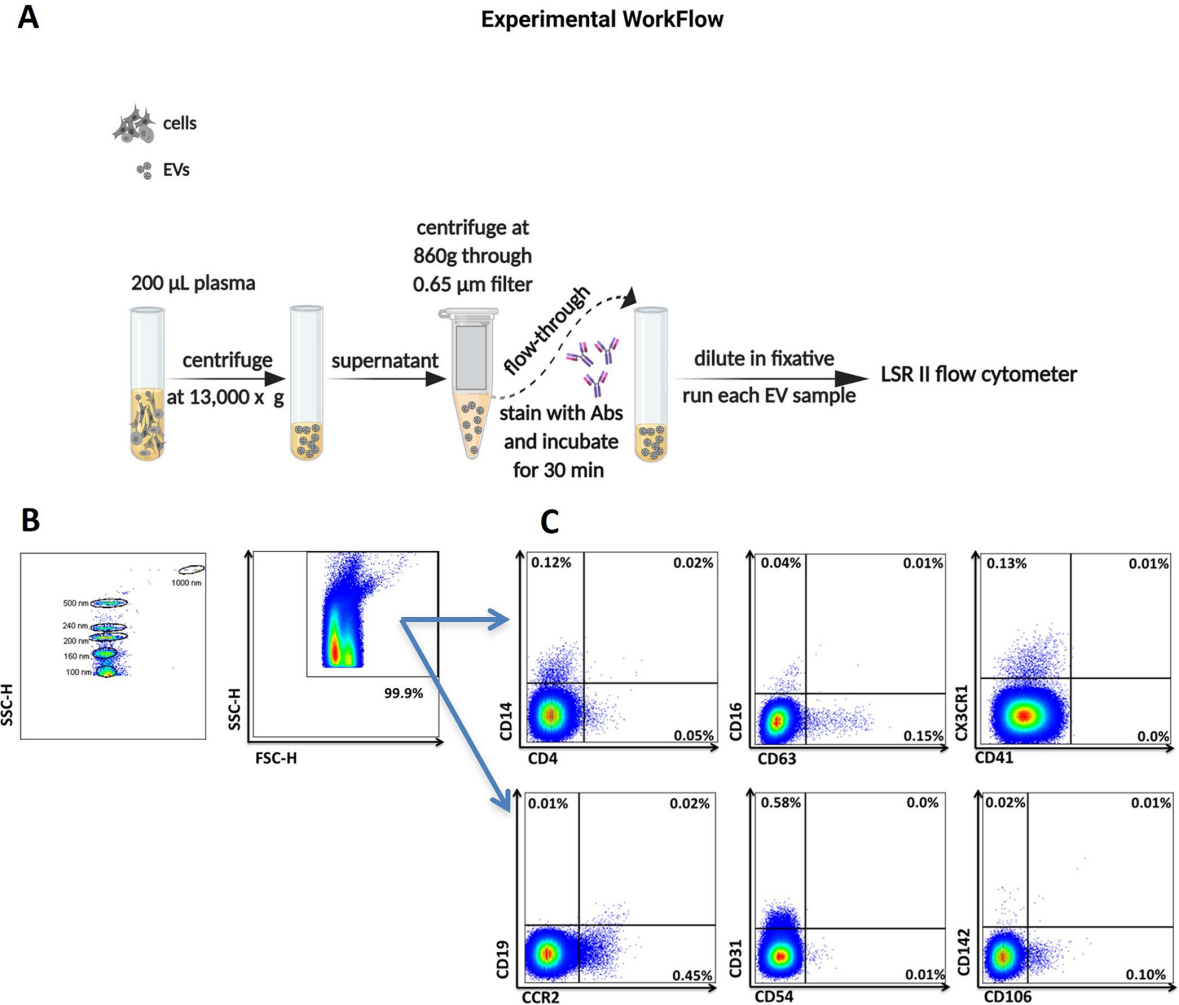
Characterizing EVs in biological samples for their relative size, absolute count, and cell of origin. (A) Schematic of the method for isolation and analysis of EVs. (B) SSC height (SSC-H) dot plot showing sensitivity to detect a mix of polystyrene beads 100 to 1,000 nm in diameter, a size range used to set EV gates on the LSRII flow cytometer. (C) Representative plots showing EVs expressing surface markers from their cell of origin.

**FIG 2:**
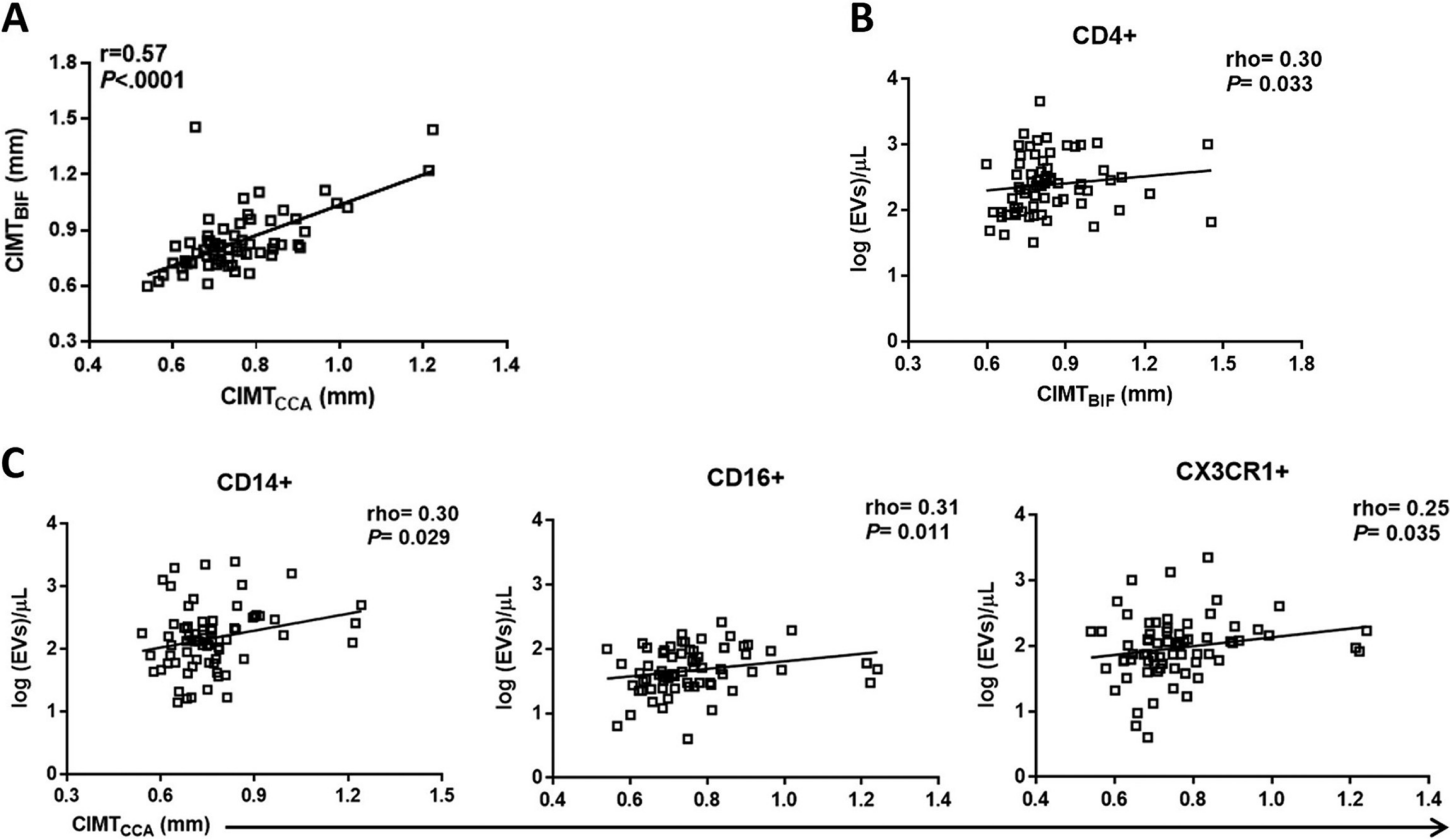
Circulating EVs expressing monocyte markers are related to carotid artery intima-media thickness in HIV-infected individuals. (A) Scatterplots demonstrating the association between the right common carotid (cIMT_CCA_) and the right carotid bifurcation (cIMT_BIF_) intima-media thickness. (B and C) Correlation of EVs expressing lymphocyte- (B) and monocyte-associated markers (C) with cIMT, evaluated using Spearman’s rank correlation coefficient test. After correcting for multiple testing with an FDR of <0.1, CD16^+^ EVs remained associated with cIMT. Dots represent individual participants.

For surface protein profiles of EVs, we examined three panels consisting of several antigens linked to lymphoid cells (CD4, CD19, and CD27), myeloid cells (CD14, CD16, CD163, CCR2, CCR5, CXCR1, CD41a, and CD62p), endothelial cells (CD31, CD54, CD106, CD141, and CD142), and multivesicular bodies (CD63). EV gates were set just below the 100-nm bead signal up to the 1,000-nm bead signal on the side scatter (SSC) channel (Fig. 1B). Representative flow cytometry plots are shown in Fig. 1C. EV phenotypes were compared from 74 ART-suppressed HIV-infected and 64 uninfected individuals. Total EV count was higher in the HIV-infected group, and EVs expressing the lymphoid markers CD19 and CD27, the myeloid markers CD14, CD16, CD163, CX3CR1, CD192, and CD195, the exosome marker CD63, and the endothelial marker CD31 were all lower in the HIV-infected group (see Fig. S1 in the supplemental material), consistent with a prior report (26). We next assessed the relationship of EV phenotypes and cIMT. There was a positive correlation between the CCA-IMT and BIF-IMT (*r* = 0.57, *P* < 0.0001) (Fig. 2A). Bivariate analysis showed a correlation between BIF-IMT but not CCA-IMT and levels of plasma EVs expressing CD4^+^ in HIV-infected individuals (*r* = 0.30, *P* = 0.033, false-discovery rate [FDR] = 0.11) (Fig. 2B). CCA-IMT correlated with EVs expressing CD14 (*r* = 0.30, *P* = 0.029, FDR = 0.10), CD16 (*r* = 0.31, *P* = 0.011, FDR = 0.04), and CX3CR1 (*r* = 0.25, *P* = 0.035, FDR = 0.11) in HIV^+^ participants (Fig. 2C). These correlations were not significant for BIF-IMT, although the direction of association was the same (data not shown). No associations were observed between circulating EVs and cIMT in the uninfected control group.

10.1128/mbio.03005-21.1 FIG S1 Comparison of EV levels in HIV-infected and uninfected participants. EVs were quantified by flow cytometry in previously frozen plasma samples; concentration was determined using TruCount tubes. Total EV counts and concentration of each subtype of EV are shown. Comparisons between HIV-infected and uninfected subjects were performed using the Mann-Whitney test. Download FIG S1, PDF file, 0.4 MB. Copyright © 2022 Marques de Menezes et al. 2022 Marques de Menezes et al. https://creativecommons.org/licenses/by/4.0/ This content is distributed under the terms of the Creative Commons Attribution 4.0 International license.

To explore whether covariates could confound the relationship between monocyte-derived EVs and cIMT, the relationship between cIMT and age, gender, BMI, HIV viremia, diabetes mellitus, smoking, prior AIDS diagnosis, and nadir CD4 count was determined. Of the potential covariates, only age significantly interacted with CCA-IMT, but not BIF-IMT. A multivariate *P* value was computed for each of the correlations between EVs and cIMT after controlling for age and other covariates. The *P* value remained significant for the correlation of CD4^+^, CD16^+^, and CX3CR1^+^ EVs with cIMT, while the association between total EV count and CD14^+^ EV count showed a *P* value of 0.08. To understand the interaction between EVs and ART regimen, current and past use of nucleoside reverse transcriptase inhibitors (NRTIs), nonnucleoside reverse transcriptase inhibitors (NNRTIs), protease inhibitors, and abacavir was correlated with monocyte-derived EV populations. Current but not past NNRTI use was associated with CD14^+^ EVs (*P* = 0.032), and current but not past protease inhibitor use was associated with CX3CR1^+^ EVs (*P* = 0.024). To explore whether ART class or abacavir usage could confound the relationship between EVs and cIMT, partial Spearman correlations were calculated and adjusted for ART. A multivariate estimate was computed for each of the correlations between EVs expressing CD4, CD14, CD16, and CX3CR1 and cIMT after controlling for ART regimens. There was a modest effect of NNRTI and protease inhibitor use on CD14^+^ and CX3CR1^+^ EVs, as the correlation between CD14^+^ EVs and cIMT changed from rho = 0.26 (*P* = 0.029) to rho = 0.23 (*P* = 0.062) after controlling for current NNRTI use. Similarly, the correlation between CX3CR1^+^ EVs and cIMT changed from rho = 0.25 (*P* = 0.035) to rho = 0.20 (*P* = 0.11) after controlling for current protease inhibitor use. Finally, a sensitivity analysis was performed to exclude the eight participants with detectable viremia. Of the monocyte-derived EVs, only the *P* value for the association between CD4^+^ EVs and cIMT increased to 0.079, though the direction of the association remained consistent (Fig. S2a).

10.1128/mbio.03005-21.2 FIG S2 Sensitivity analysis of effect of viremia on cIMT and CD4^+^ EVs and soluble biomarkers. Scatterplots are shown for the total HIV^+^ population (*n* = 74) as well as the aviremic (*n* = 66) and viremic (*n* = 8) participants. Correlation was evaluated using Spearman’s rank correlation coefficient test between cIMT and EVs expressing CD4 (a), VCAM-1 (b), ICAM-1 (c), CRP (d), and SAA (e). Dots represent individual participants. Download FIG S2, PDF file, 0.2 MB. Copyright © 2022 Marques de Menezes et al. 2022 Marques de Menezes et al. https://creativecommons.org/licenses/by/4.0/ This content is distributed under the terms of the Creative Commons Attribution 4.0 International license.

### Relationship between levels of EV phenotypes and soluble inflammatory markers.

**FIG 3:**
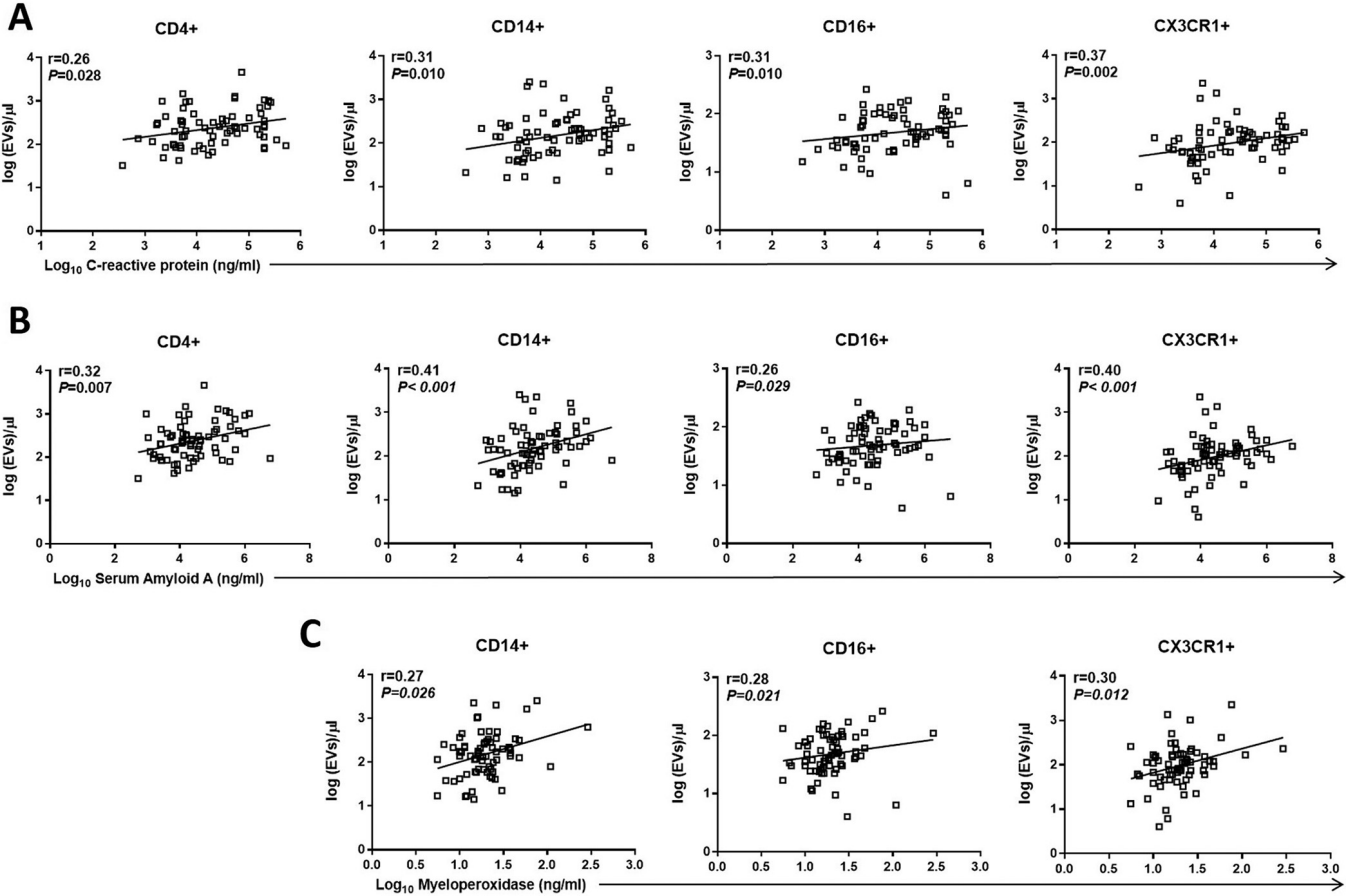
EVs expressing monocyte-associated markers are correlated with plasma soluble inflammatory markers. Scatterplots demonstrating the relationship between levels of EVs expressing monocyte-associated markers and levels of soluble C-reactive protein (A), serum amyloid A (B), and myeloperoxidase (C). Spearman correlation coefficient (*R*) and significance (*P*) are indicated in the corresponding boxes. These results remained significant after correcting for multiple testing with an FDR of <0.1. Dots represent individual patients.

We next explored associations between EV phenotypes and soluble inflammatory markers from 74 cART-treated participants. The mean concentration of soluble biomarkers is shown in Table S1. We found an association between levels of C-reactive protein (CRP) and EVs expressing CD4 (*r* = 0.26, *P* = 0.028), CD14 (*r* = 0.31, *P* = 0.010), CD16 (*r* = 0.31, *P* = 0.010), and CX3CR1 (*r* = 0.37, *P* = 0.002) (Fig. 3A). There was a significant correlation between levels of serum amyloid A (SAA) and CD4^+^ EVs (*r* = 0.32, *P* = 0.007), CD14^+^ EVs (*r* = 0.41, *P* < 0.001), CD16^+^ EVs (*r* = 0.26, *P* = 0.029), and CX3CR1^+^ EVs (*r* = 0.40, *P* < 0.001) (Fig. 3B). We also observed a correlation between levels of myeloperoxidase (MPO) and CD14^+^ EVs (*r* = 0.27, *P* = 0.026), CD16^+^ EVs (*r* = 0.28, *P* = 0.021), and CX3CR1^+^ EVs (*r* = 0.30, *P* = 0.012) (Fig. 3C). Levels of interleukin-6 (IL-6) trended positively with EVs expressing CD14 (*r* = 0.24, *P* = 0.047), CD16 (*r* = 0.24, *P* = 0.047), and CX3CR1 (*r* = 0.26, *P* = 0.030) (FDR > 0.1). No correlation was noted between cIMT and soluble inflammatory markers. In a sensitivity analysis we excluded the eight viremic participants, and levels of VCAM-1, ICAM-1, CRP, and SAA were associated with cIMT in the aviremic HIV-infected participants (Fig. S2b to e).

10.1128/mbio.03005-21.4 TABLE S1 Results are reported as mean values ± standard deviations (SD). Abbreviations: MCP-1, monocyte chemoattractant protein-1; IFN-γ, interferon gamma; VCAM-1, vascular cell adhesion molecule-1; ICAM-1, intercellular cell adhesion molecule-1; VEFG, vascular endothelial growth factor; MPO, myeloperoxidase; MMP-9, matrix metallopeptidase 9; PAI-1, plasminogen activator inhibitor-1; CRP, C-reactive protein; SAA, serum amyloid A; SAP, serum amyloid protein. Download Table S1, PDF file, 0.03 MB. Copyright © 2022 Marques de Menezes et al. 2022 Marques de Menezes et al. https://creativecommons.org/licenses/by/4.0/ This content is distributed under the terms of the Creative Commons Attribution 4.0 International license.

**FIG 4:**
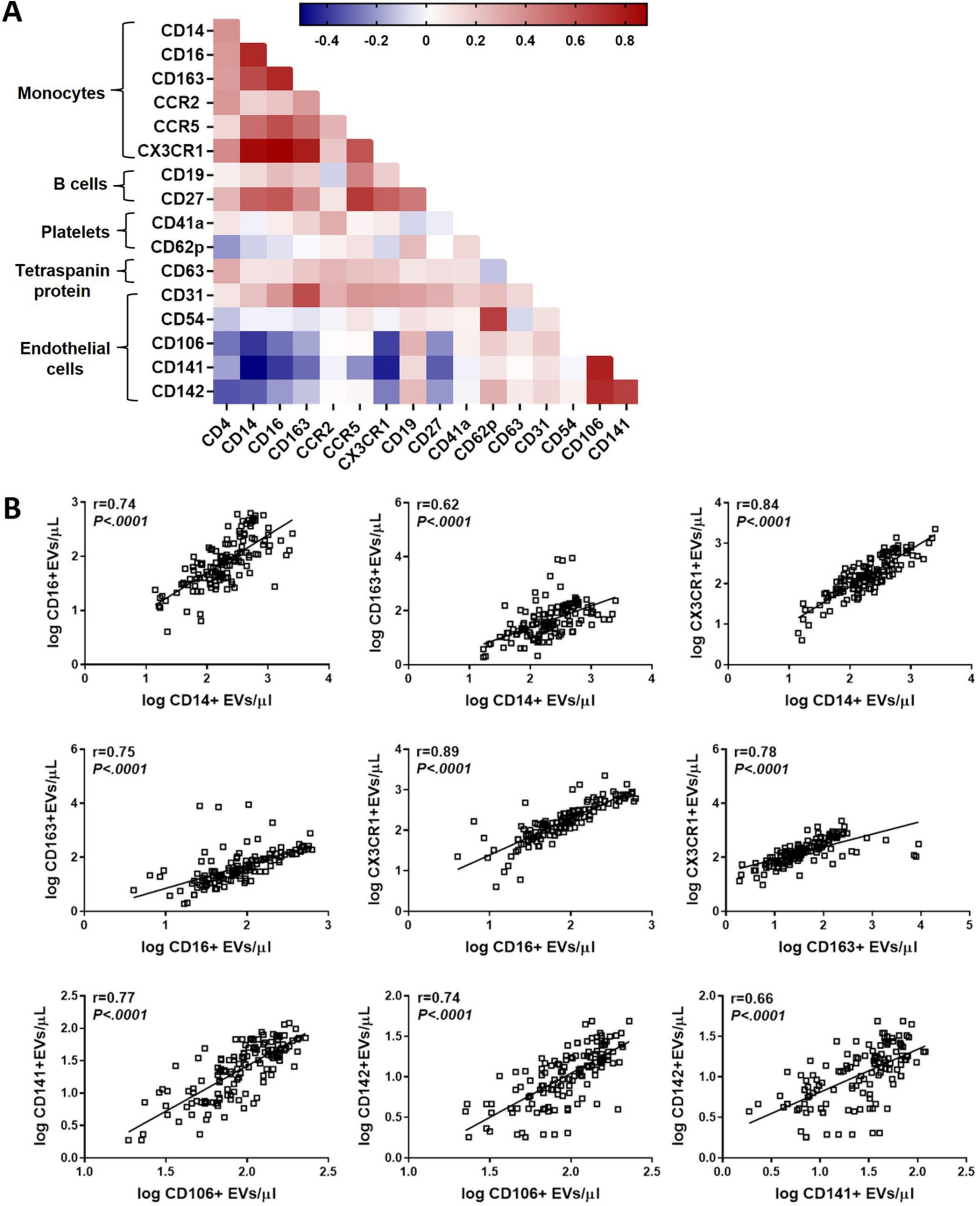
Spearman rank correlations between all EV subtypes. (A) Spearman correlation matrix showing the relationship between all EV subtypes based on their cellular origin. Positive correlations are displayed in red. (B) Scatterplots demonstrating the strong correlations of surface markers with each other on EVs. These results remained significant after correcting for multiple testing with an FDR of <0.1. Dots represent individual patients.

Finally, to further evaluate generalizability of our data, we determined the relationship between all EV subtypes based on their surface marker phenotype in plasma samples from all participants (Fig. 4). Using Spearman’s rank correlation, we found a significant positive correlation of monocyte-associated markers with each other on EVs: CD14, CD16, CD163, and CX3CR1. There was a relationship between endothelial markers derived from EVs: CD106, CD141, and CD142. No significant correlation was found between cell surface markers known to reside on different cells. These results remained significant when the groups were tested separately (FDR < 0.1).

### EV activity on endothelial cell death.

**FIG 5:**
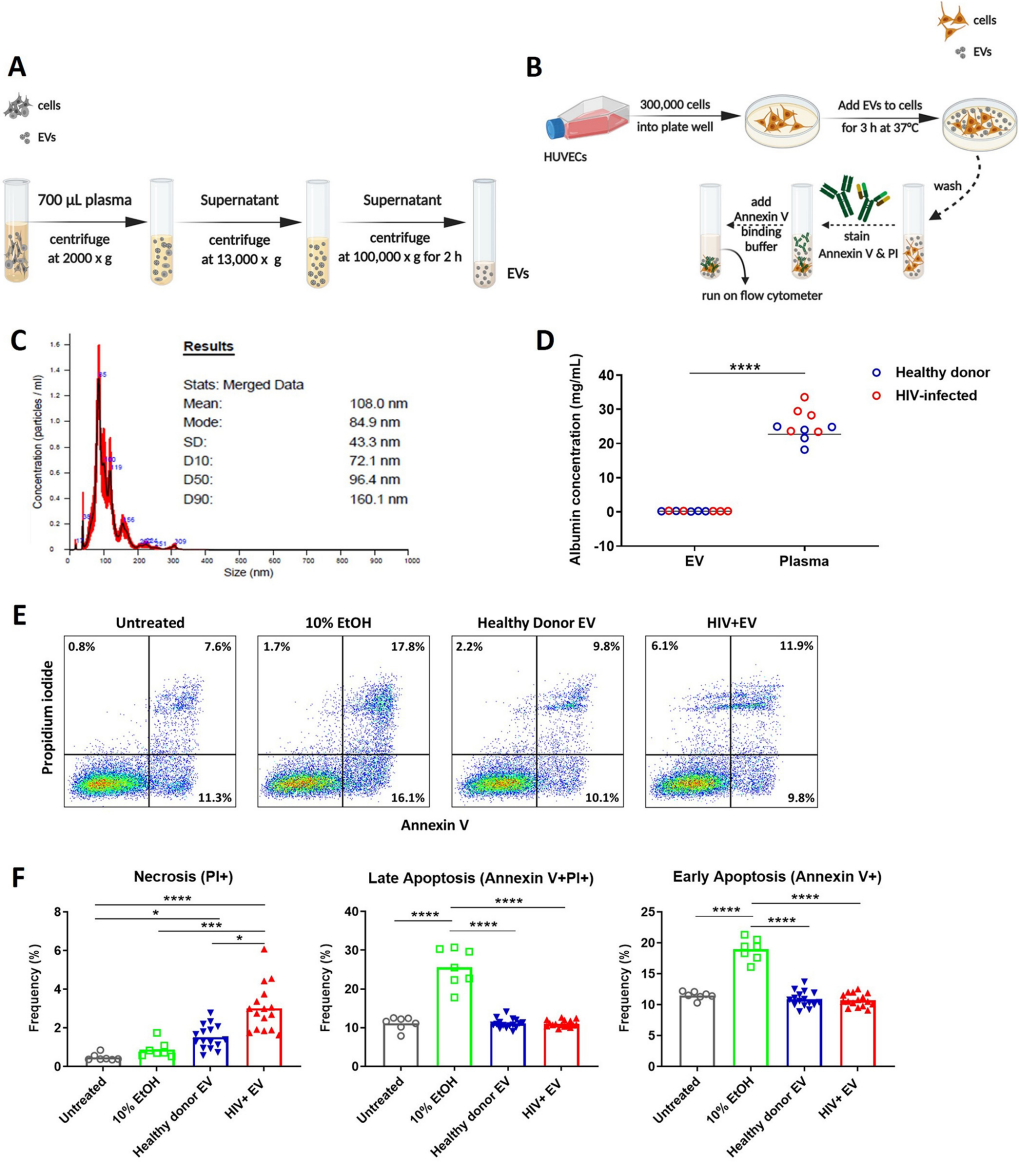
Apoptosis and necrosis rates of HUVECs after cell exposure to EVs. (A and B) Schematic of the EV isolation method (A) and apoptosis assay (B). (C) Representative graph of nanoparticle tracking analysis showing the EV particle-size distribution generated by ultracentrifugation of plasma. EV preparations from three uninfected participants were measured, each with similar size distribution. (D) Levels of the non-EV-associated protein albumin in the original plasma and EV samples were determined by ELISA (*n* = 5 HIV-uninfected donors and 5 HIV-infected participants). (E) Representative flow cytometry plots for cells without treatment and treated with EVs or 10% ethanol (EtOH). (F) *P* values were determined by using one-way analysis of variance (ANOVA) and Tukey’s multiple-comparison *post hoc* test. *, *P* < 0.05; ***, *P* < 0.001; ****, *P* < 0.0001.

To determine if EVs have direct effects on HUVECs, we measured EV-induced apoptosis and necrosis using annexin V and propidium iodide (PI) (Fig. 5A and B). We tested four groups, including control untreated HUVECs, HUVECs treated with 10% ethanol (10% EtOH), HUVECs treated with EVs derived from 16 HIV-infected individuals (HIV^+^EV), and HUVECs treated with EVs from 16 uninfected controls (healthy donor EV). Characterization of EVs using nanoparticle tracking analysis (NTA) demonstrated that EVs generated by ultracentrifugation contained predominantly smaller particles with a mean diameter of 108 nm (Fig. 5C). Analysis of the amount of albumin determined by enzyme-linked immunosorbent assay (ELISA) revealed that the EV samples obtained by ultracentrifugation were on average 93-fold lower in albumin than the original plasma, implying that the EV fraction was substantially depleted of plasma proteins (Fig. 5D). Representative flow cytometry plots for HUVECs that were untreated or treated with EVs or 10% EtOH are presented in Fig. 5E. The results indicated that initial apoptosis (annexin V^+^ PI^−^) and late apoptosis (annexin V^+^ PI^+^) were significantly increased in the 10% EtOH treatment group compared with the untreated cells and EVs. Interestingly, necrosis (annexin V^−^ PI^+^) was significantly increased after HUVEC exposure to EVs from HIV-infected individuals compared to that after exposure to EVs from the uninfected control group, 10% EtOH control, and untreated cells (Fig. 5F). In summary, the results indicated that the EV fraction from HIV-infected individuals led to increased endothelial cell death via necrosis.

Analyses of EVs generated from plasma of HIV-infected individuals are complicated by the difficulty in separating EVs from virions by available approaches (differential ultracentrifugation and/or velocity gradient) because of the similarity of structure and biophysical properties (27, 28). To address the question of whether EVs derived from plasma with high HIV RNA levels may lead to greater necrotic cell death, we additionally measured EV apoptosis-inducing activity from HIV-infected individuals with high viral load (>500,000 copies/mL) versus low viral load (<50 copies/mL). One million EVs derived from approximately 10 μL of plasma were added to HUVECs for 3 h prior to apoptosis and necrosis quantitation. HUVECs showed increased necrosis after exposure to the EVs from HIV-infected individuals with high viral load compared to that after exposure to the EVs from individuals on virologically suppressive cART and uninfected controls (*P* < 0.01). There was no significant difference in early apoptosis or late apoptosis rates between the EV fractions from HIV-infected individuals with low viral load and the fractions from those with high viral load, but there was a significant difference in early apoptosis rate between the uninfected controls and HIV-infected individuals with high viral load (Fig. S3).

10.1128/mbio.03005-21.3 FIG S3 Effect of EVs derived from HIV-infected individuals with a high viral load on HUVEC death. (a) Representative gating by flow cytometry for HUVECs untreated or treated with 10% ethanol (EtOH), EVs from HIV-uninfected individuals, EVs from HIV-infected individuals on suppressive antiretroviral therapy (ART), and EVs from HIV-infected individuals with high viral loads. (b) Comparisons were evaluated using the Kruskal-Wallis test and Dunn’s multiple-comparison *post hoc* test. Statistical significance is indicated as follows: *, *P* < 0.05; ***, *P* < 0.001; ****, *P* < 0.0001. Download FIG S3, PDF file, 0.5 MB. Copyright © 2022 Marques de Menezes et al. 2022 Marques de Menezes et al. https://creativecommons.org/licenses/by/4.0/ This content is distributed under the terms of the Creative Commons Attribution 4.0 International license.

## DISCUSSION

To the best of our knowledge, this study provides the first evidence that levels of EVs expressing CD16 are associated with cIMT in HIV-infected individuals on virologically suppressive cART. Notably, trends toward an association with cIMT were observed for EVs expressing other monocyte-associated markers, including CD14 and CX3CR1, in HIV-infected individuals on cART. Furthermore, we also show here that EVs or virions derived from HIV-infected individuals on stable cART induced endothelial cell death via necrosis.

Given that other studies have demonstrated an association between cIMT and future cardiovascular disease, one interpretation of our data is that plasma monocyte-derived vesicles, as indicated by CD16, may be part of HIV-driven immune activation and inflammation, which would subsequently drive the increased risk of CVD seen in chronically HIV-infected adults on virologically suppressive cART (12, 29). Interestingly, in another study immunophenotyping peripheral blood mononuclear cells (PBMCs) from the Hawaii Aging with HIV Cardiovascular Disease (HAHC-CVD) cohort, nonclassical monocytes were associated with cIMT in HIV-infected individuals on stable cART independent of Framingham risk score (12), a finding similar to what we observed in EVs carrying CD16 on their surface. These results imply that using a plasma or serum sample could give insight into cellular phenotype without access to PBMC samples. Given the ease and lower cost of obtaining plasma and serum samples, this could represent a path to explore cellular studies that would not be possible in cohorts without dedicated PBMC banking. Another study performed by Eikendal et al. (30) reported an association between EV-derived monocyte markers and cIMT in healthy young children. Although we observed that levels of cIMT were similar between HIV-infected individuals on stable cART and uninfected controls, no associations between EV phenotypes and cIMT were noted in the uninfected control group.

Notably, none of the plasma soluble markers of inflammation evaluated showed a significant association with cIMT, consistent with a prior study from the HAHC-CVD cohort (10). In contrast, it has been reported that levels of monocyte chemoattractant protein and C-reactive protein were independently associated with cIMT in treated HIV infection (31, 32). Although the plasma soluble inflammatory biomarkers were not related to cIMT, we observed that levels of serum amyloid A, C-reactive protein, and myeloperoxidase were significantly correlated with EVs expressing CD14, CD16, and CX3CR1 in HIV-infected individuals on cART. Taken together, these results imply that plasma EV-derived monocytes may inform cardiovascular risk through inflammation and immune activation among individuals on cART with viral suppression. The pathophysiological mechanism underlying this relationship remains undefined, though candidate mechanisms include EV-associated cytokines (33), viral proteins, microRNA (miRNA), bioactive lipids, or other EV cargo (reviewed in reference 34).

We found that the EV-containing fraction of plasma derived from HIV^+^ cART-suppressed individuals induces endothelial cell death via necrosis. Additionally, the EV fraction from individuals with high viral load had higher apoptosis-inducing activity. A simple explanation would be that HIV virions directly induce apoptosis in HUVECs and that contaminating HIV virions contributed to the increase in necrosis seen. However, the data shown in Fig. 5F were drawn from HIV-infected participants with viral loads of <50 RNA copies/mL. Only 10 μL of starting material was introduced into the assay, which would be the equivalent of 0.5 RNA copies per experiment. Given that each virion contains two RNA copies, that would be the equivalent of 0.25 virions per experiment (35). It is much more likely that the 1 million EVs were responsible for the excess necrosis seen in the primary study. However, for individuals with higher viral load (∼500,000 copies/mL), the increased necrosis seen compared to individuals with low viral load could indeed be due to virions themselves (see Fig. S1 in the supplemental material). EVs generated by HIV-infected cells can be transferred rapidly to uninfected cells (36). Nef-expressing or HIV-infected cells secrete EVs that can deliver signals in *trans* and induce apoptosis in bystander cells (37). Aqil et al. (38) reported that HIV Nef-expressing EVs could regulate proinflammatory cytokines and target genes involved in several pathways including apoptosis, mitogen-activated protein kinase signaling, and transforming growth factor β (TGF-β) signaling.

Our data show that EVs and potentially virions can induce necrosis in HUVECs, which is consistent with prior work showing induction of caspase-3 after exposure to EVs from HIV-infected individuals (22). It is tempting to speculate about pathways and gene targets that may have contributed to our observed results, particularly miRNAs, given that they are highly enriched in exosomes. The literature suggests that monocyte-derived EVs can promote phosphorylation of intracellular signaling pathways and induce the expression of cell adhesion molecules on endothelial cells in an IL-1β-dependent manner (39). Aharon et al. (18) reported that monocyte EVs generated by costimulation with lipopolysaccharide (LPS) and calcium ionophore induce apoptosis and tissue factor expression in endothelial cells. EVs derived from the supernatant of activated, and potentially atherogenic, CD4^+^ T lymphocytes enhance cholesterol accumulation in cultured monocytes in a phosphatidylserine receptor-dependent manner (40). Interestingly, a recent study reported that distinct EV populations derived from human T cell lymphotropic virus type 1 (HTLV-1)-infected cells may induce proinflammatory cytokine secretion, angiogenesis, cellular damage, and enhance viral spread (41). Plasma-derived EVs contain higher levels of TGF-β1 in HIV-infected individuals with pulmonary hypertension and have the ability to promote endothelial and smooth muscle cell dysfunction (42). Given that endothelial activation markers correlate with cIMT in HIV-infected patients (43) and that EVs from HIV-infected subjects can activate HUVECs (22), our data showing that EVs can cause necrosis of HUVECs would be consistent with a model of EV-induced endothelial cell damage contributing to atherosclerosis.

Although our results support the role of circulating EVs as a marker of HIV-associated cardiovascular disease, our study is limited by its targeted approach and relatively small cross-section study of 74 HIV-infected individuals. Our study population may be distinct from other clinical populations, as our study participants had a low risk of CVD, normal cIMT values, a median CD4^+^ T cell count of nearly 500 cells/μL, and well-controlled viremia. An additional study limitation was that our covariates were measured only at baseline, which limited our ability to evaluate the effects of changes in risk factors and disease characteristics on cIMT progression. Further investigations using a larger HIV-CVD cohort and the use of a validation cohort are needed to better characterize the EVs and confirm the relationship of EVs expressing monocyte markers with cIMT in order to use it as a circulatory biomarker of HIV-CVD.

Understanding the role played by EVs in the pathogenesis of HIV-associated cardiovascular disease remains a crucial research goal. Further investigation of these EVs to elucidate their potential as biomarkers of and potential immunotherapeutic targets in the pathogenesis of CVD in the setting of treated HIV disease is warranted.

## MATERIALS AND METHODS

### Study participants.

This study utilized archived plasma samples from the Hawaii Aging with HIV Cardiovascular Disease (HAHC-CVD) study, a 5-year longitudinal natural history cohort study designed to investigate the role of immune activation and inflammation in the pathogenesis of CVD in HIV-infected individuals on cART (44). HIV-negative adult participants were enrolled as the control comparison group from the HAHC-CVD study and were matched for age, gender, ethnicity, body mass index (BMI), and comorbidities (45). At enrollment demographic information and general medical and CVD history were obtained. HIV-1 viral load, CD4 T cell count, blood pressure, height, weight, and BMI were measured (44, 45). The HAHC-CVD study was approved by the University of Hawaii Committee on Human Subjects, and informed consent was obtained from all participants. Additional plasma samples from 6 HIV-infected individuals with high viral load (>500,000 RNA copies/mL) and 6 with low viral load (<50 RNA copies/mL) from the External Quality Assurance Program Oversight Laboratory (EQAPOL) study (46, 47) were utilized for the functional experiments.

### Plasma extracellular vesicle processing.

Blood samples were collected into EDTA tubes, and separated plasma was stored at −80°C. For the characterization of EV surface markers, 200 μL of plasma was centrifuged at 13,000 × *g*, after which the supernatant was then centrifuged through 0.65-μm-pore-size filters (Millipore) for 10 min at 860 × *g* or until most supernatant had passed through. The flowthrough was then collected and used to measure EV concentration and phenotype (Fig. 1A) (48). Purified EVs for functional experiments were isolated using differential centrifugation of 700 μL plasma with an initial speed of 2,000 × *g* for 10 min, followed by spinning 600 μL of the supernatant at 13,000 × *g* for 30 min at 4°C. Next, 500 μL of supernatant was ultracentrifuged at 100,000 × *g* for 2 h at 4°C. EV pellets were resuspended in 500 μL phosphate-buffered saline (PBS) prefiltered with a 0.22-μm-pore-size Ultrafree MC-GV centrifugal filter (Millipore), aliquoted, and stored at −80°C.

### EV immunophenotyping by flow cytometry.

Plasma samples were stained using fluorochrome-conjugated monoclonal antibodies in three separate panels, purchased from BioLegend unless otherwise noted: lymphocytes CD4-BV421 (clone: OK-T4), CD19-Alexa 700 (SJ25C1), and CD27-peridinin chlorophyll protein (PerCP)/Cy5.5 (M-T271); monocytes CD14-allophycocyanin (APC) (63D3), CD16-phycoerythrin (PE) (3G8), CD163-PECy7 (GHI6I), CCR2 ([C-C chemokine receptor type 2]-V421 [K036C2]), CCR5 ([C-C chemokine receptor type 5]-PE [3A9; BD Biosciences]), and CX3CR1 ([CX3C chemokine receptor 1]-fluorescein isothiocyanate [FITC] [2A9.1]); platelets CD41a-PerCP/Cy5.5 (HIP8) and CD62p-FITC (AK-4; BD Biosciences); tetraspanin protein CD63-BV421 (H5C6); and endothelial CD31-PECy7 (WM59), CD54-Pacific blue (HA58), CD106-PE (STA), CD141-PerCP/Cy5.5 (M80), and CD142-APC (NY2). Antibodies were titrated for optimal concentration. Isotype controls and fluorescence-minus-one (FMO) controls were used to measure background fluorescence. Antibodies were filtered using a 0.22-μm centrifugal filter to remove aggregates. Antibodies (1 to 5 μL) were added to 10 μL of EVs and incubated at 4°C for 30 min. EVs were diluted in 0.22-μm-filtered PBS containing 2.8% formaldehyde (BD stabilizing fixative) to appropriate dilutions to avoid swarm detection.

Samples were acquired on an LSRII flow cytometer (BD). Forward scatter (FSC) and side scatter (SSC) were set to the maximum voltages of 500 to 600 and 300 to 400, respectively. The FSC and SSC detectors were set to a triggering threshold of 200 to minimize machine noise. Gates were established using 0.1-μm (Invitrogen) to 1-μm beads (Megamix, 0.16, 0.2, 0.24, and 0.5 μm; Spherotech, 1 μm) (14, 49). Samples were acquired for 60 s at a low flow rate (8 to 12 μL/min). TruCount tubes (BD Biosciences) were used to measure the concentration of EVs. Analysis was performed using FlowJo software (version 10.7.1; BD).

### Nanoparticle tracking analysis (NTA) and albumin quantification.

The concentration of purified EVs for functional experiments was evaluated using a NanoSight NS300 instrument (Malvern) configured with a syringe pump flow rate of 30 and a 405-nm laser. The samples were diluted 1:1,000 in 0.22-μm-filtered PBS. Five videos were recorded at camera level 14, acquisition time 30 s, and a detection threshold of 5. Data analysis was performed with NTA v3.3 software. To assess EV purity, albumin was measured by ELISA (Bethyl) in plasma and purified EV samples (50, 51).

### Cell culture.

Human umbilical cord vein endothelial cells (HUVECs) were obtained from the American Type Culture Collection (ATCC). HUVECs were cultured in vascular cell basal medium (PCS100030; ATCC) supplemented with vesicle-free endothelial cell growth kit components (PCS100041; ATCC) with the addition of 1% penicillin-streptomycin (PCS999002; ATCC) and 2% exosome-free fetal bovine serum (FBS) (EXO-FBSHI-250A-1; Systems Biosciences). HUVECs at passage 3 were cultured in 6-well plates until they reached 80% confluence.

### Apoptosis measurement.

Purified EVs were counted using TruCount tubes (BD Biosciences) on an LSRII flow cytometer. HUVECs were incubated with 1 million purified EVs in standard vesicle-free endothelial growth medium. For positive controls cells were treated with 10% ethanol (EtOH) (52). After 3 h, cell supernatants were mixed with the trypsinized cells to include detached dead cells in the analysis, and cells were stained with annexin V-APC (A35110; Life Technologies) and PI (421301; BioLegend) in annexin V binding buffer (V13246; Life Technologies). Cells were analyzed by flow cytometry immediately after treatment and staining.

### Multiplex immunoassay.

Aliquots of plasma from 74 HIV-infected individuals on cART were used for multiplex biomarker analysis using Milliplex human cardiovascular disease panels (EMD Millipore, USA). Soluble biomarkers assessed included sE-selectin, sVCAM-1, sICAM-1, matrix metalloprotease 9 (MMP-9), myeloperoxidase (MPO), plasminogen activator inhibitor-1 (tPAI-1), C-reactive protein (CRP), serum amyloid A (SAA), serum amyloid P (SAP), monocyte chemoattractant protein-1 (MCP-1), vascular endothelial growth factor (VEGF), IL-1β, IL-6, IL-8, IL-10, tumor necrosis factor alpha (TNF-α), and gamma interferon (IFN-γ). Standards and samples were measured in duplicate. Samples were acquired on a Luminex 200 instrument (R&D Systems, Inc.). Bio-Plex Manager 6.1 software (Bio-Rad Laboratories, Inc.) was used for data processing.

### Carotid artery intima-media thickness measurement.

High-resolution B-mode ultrasound images of the right carotid artery were obtained to measure the cIMT of the far wall of the distal common carotid artery (CCA) and bifurcation (BIF) with automated edge detection as previously reported (45). cIMT values were defined as normal when <0.8 mm, as suspect for vascular disease when 0.8 to 0.99 mm (53, 54), and as pathological when ≥1.0 mm (55). Ultrasound images were acquired at the Queen’s Medical Center in Honolulu, HI, and analyzed at the University of Southern California Atherosclerosis Research Unit Core Imaging and Reading Center.

### Statistical analysis.

Data were log_10_ transformed to improve normality before further analyses were performed if their distributions were skewed. The Shapiro-Wilk test was used to determine normality. The nonparametric Mann-Whitney test was used for unpaired comparisons. Correlations between variables were performed by Spearman’s rank correlation coefficient. Functional assay data were compared using one-way analysis of variance (ANOVA) followed by Tukey’s multiple-comparison *post hoc* test as indicated, assuming normal distribution unless otherwise noted. Values of *P* ≤ 0.05 were considered statistically significant. All *P* values were two-sided, and the false-discovery rate (FDR) was calculated and a value of <0.1 was considered significant (56). Univariate analyses were conducted in Prism 9.0 (GraphPad Software). Monocyte-derived EV levels and cIMT were compared to ART class (NRTI, NNRTI, and PI) and abacavir usage using the Wilcoxon test. To perform multiple regression analysis, partial Spearman correlations were calculated and adjusted for covariates. The correlations between EVs or monocyte phenotype and cIMT were adjusted for potential covariates. The partial Spearman analyses were performed using R (PResiduals_1.0-1).
