# Supplementary material for: Plasma CD16+ Extracellular Vesicles Associate with Carotid Artery Intima-Media Thickness in HIV+ Adults on Combination Antiretroviral Therapy
Source: mBio. 2022 Apr 18;13(3):e03005-21. doi: 10.1128/mbio.03005-21 (PMC9239192; doi:10.1128/mbio.03005-21)
Supplement: FIG S1 [file mbio.03005-21-s0002.pdf]

# Figure S1

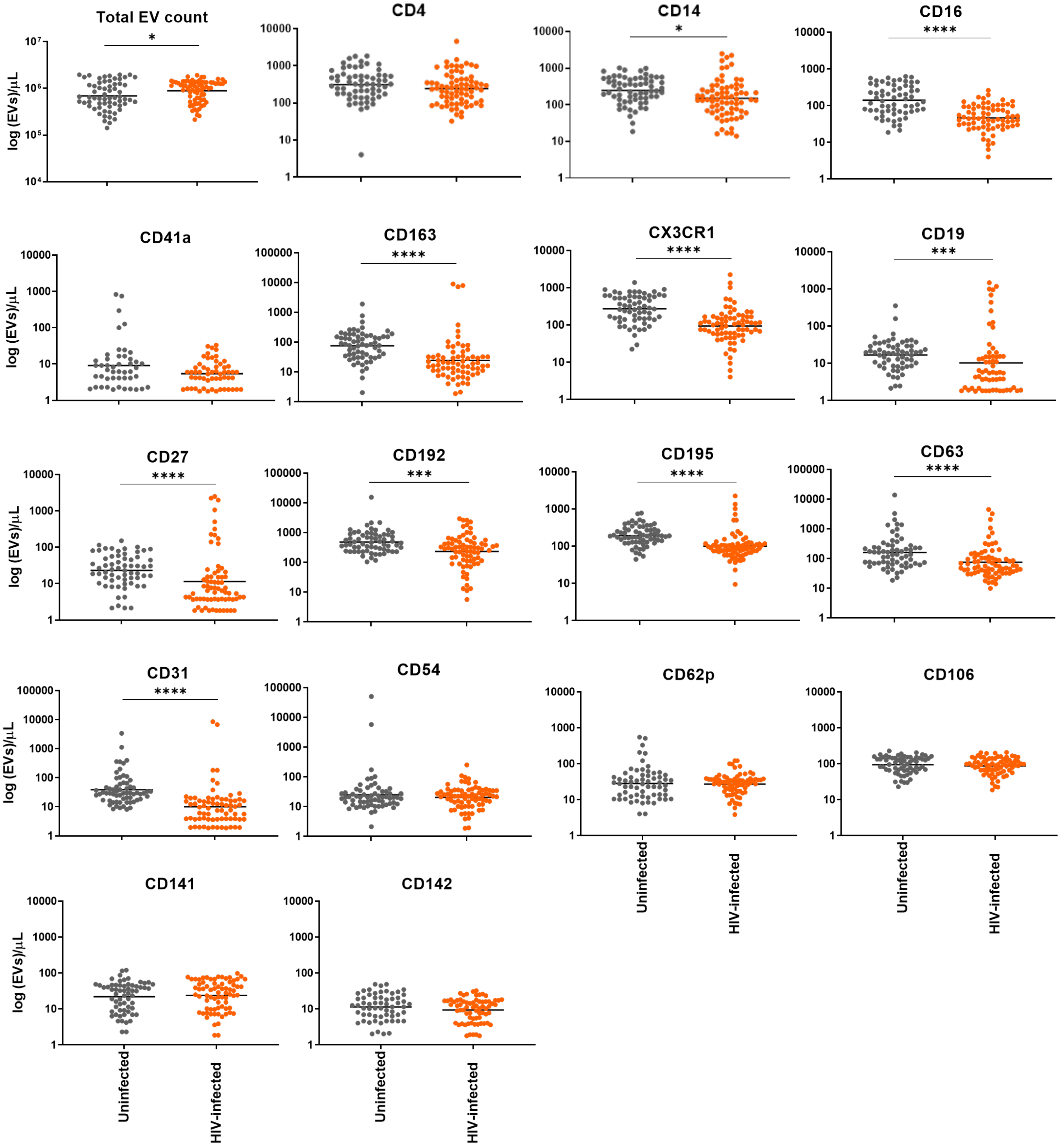


**Figure S1: Comparison of EV levels in HIV-infected and uninfected participants.** EVs were quantified by flow cytometry in previously frozen plasma samples; concentration was determined using TruCount tubes. Total EV counts and concentration of each subtype of EV are shown. Comparisons between HIV-infected and uninfected subjects was performed using the Mann-Whitney test.
