# Supplementary material for: Plasma CD16+ Extracellular Vesicles Associate with Carotid Artery Intima-Media Thickness in HIV+ Adults on Combination Antiretroviral Therapy
Source: mBio. 2022 Apr 18;13(3):e03005-21. doi: 10.1128/mbio.03005-21 (PMC9239192; doi:10.1128/mbio.03005-21)
Supplement: FIG S2 [file mbio.03005-21-s0003.pdf]

Figure S2

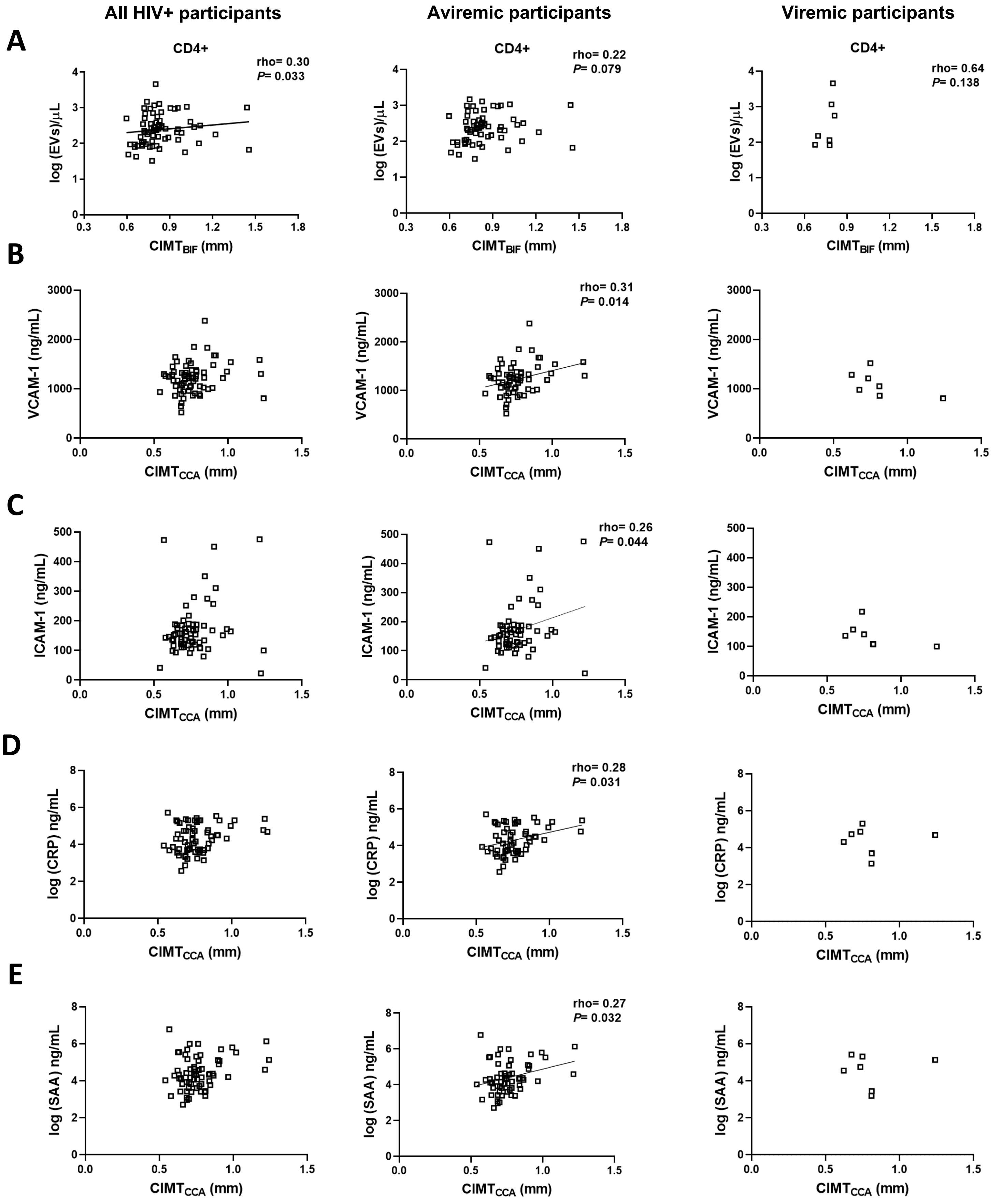

Figure S2: Sensitivity analysis of effect of viremia on cIMT and CD4+ EVs and soluble biomarkers. Scatter plots are shown for the total HIV+ population (n=74) as well as the aviremic (n=66) and viremic (n=8) participants. Correlation was evaluated using Spearman's rank correlation coefficient test is between cIMT and (a) EVs expressing CD4, (b) VCAM-1, (c) ICAM-1, (d) CRP, and (e) SAA. Dots represent individual participants.
