# Supplementary material for: Plasma CD16+ Extracellular Vesicles Associate with Carotid Artery Intima-Media Thickness in HIV+ Adults on Combination Antiretroviral Therapy
Source: mBio. 2022 Apr 18;13(3):e03005-21. doi: 10.1128/mbio.03005-21 (PMC9239192; doi:10.1128/mbio.03005-21)
Supplement: TABLE S1 [file mbio.03005-21-s0001.pdf]

**Table S1: Soluble biomarkers of HIV-infected participants.**

| Participants characteristic (*N* = 74) | Value (mean ± SD) |
|---|---|
| IL-1b (pg/ml) | 0.3 ± 0.3 |
| IL-6 (pg/ml) | 2.7 ± 5.5 |
| IL-8 (pg/ml) | 4.6 ± 3.8 |
| IL-10 (pg/ml) | 4.8 ± 12.2 |
| TNF-α (pg/ml) | 4.0 ± 4.0 |
| MCP-1 (pg/ml) | 154 ± 55.8 |
| IFN-γ (ng/ml) | 1.3 ± 1.8 |
| VCAM-1 (ng/ml) | 1220 ± 302.2 |
| ICAM-1 (ng/ml) | 167.8 ± 83.5 |
| VEGF (pg/ml) | 43.8 ± 47.1 |
| MP0 (ng/ml) | 26.1 ± 35.5 |
| MMP-9 (ng/ml) | 78.5 ± 66.5 |
| PAI-1 (ng/ml) | 104.6 ± 39.3 |
| CRP (ng/ml) | 68.2 ± 99.2 |
| SAA (ng/ml) | 206 ± 748.2 |
| SAP (ng/ml) | 187.8 ± 222.3 |
| E-selectin (pg/ml) | 48.5 ± 42.0 |

Note: Results are reported as mean values ± SD. Abbreviation: MCP-1, monocyte chemoattractant protein-1; IFN-γ, interferon-gamma; VCAM-1, vascular cell adhesion molecule-1; ICAM-1, intercellular cell adhesion molecule-1; VEFG, vascular endothelial growth factor; MPO, myeloperoxidase; MMP-9, matrix metallopeptidase 9; PAI-1, plasminogen activator inhibitor-1; CRP, C-reactive protein; SAA, serum amyloid A; SAP, serum amyloid protein.
