# Supplementary material for: Plasma CD16+ Extracellular Vesicles Associate with Carotid Artery Intima-Media Thickness in HIV+ Adults on Combination Antiretroviral Therapy
Source: mBio. 2022 Apr 18;13(3):e03005-21. doi: 10.1128/mbio.03005-21 (PMC9239192; doi:10.1128/mbio.03005-21)
Supplement: FIG S3 [file mbio.03005-21-s0004.pdf]

Figure S3

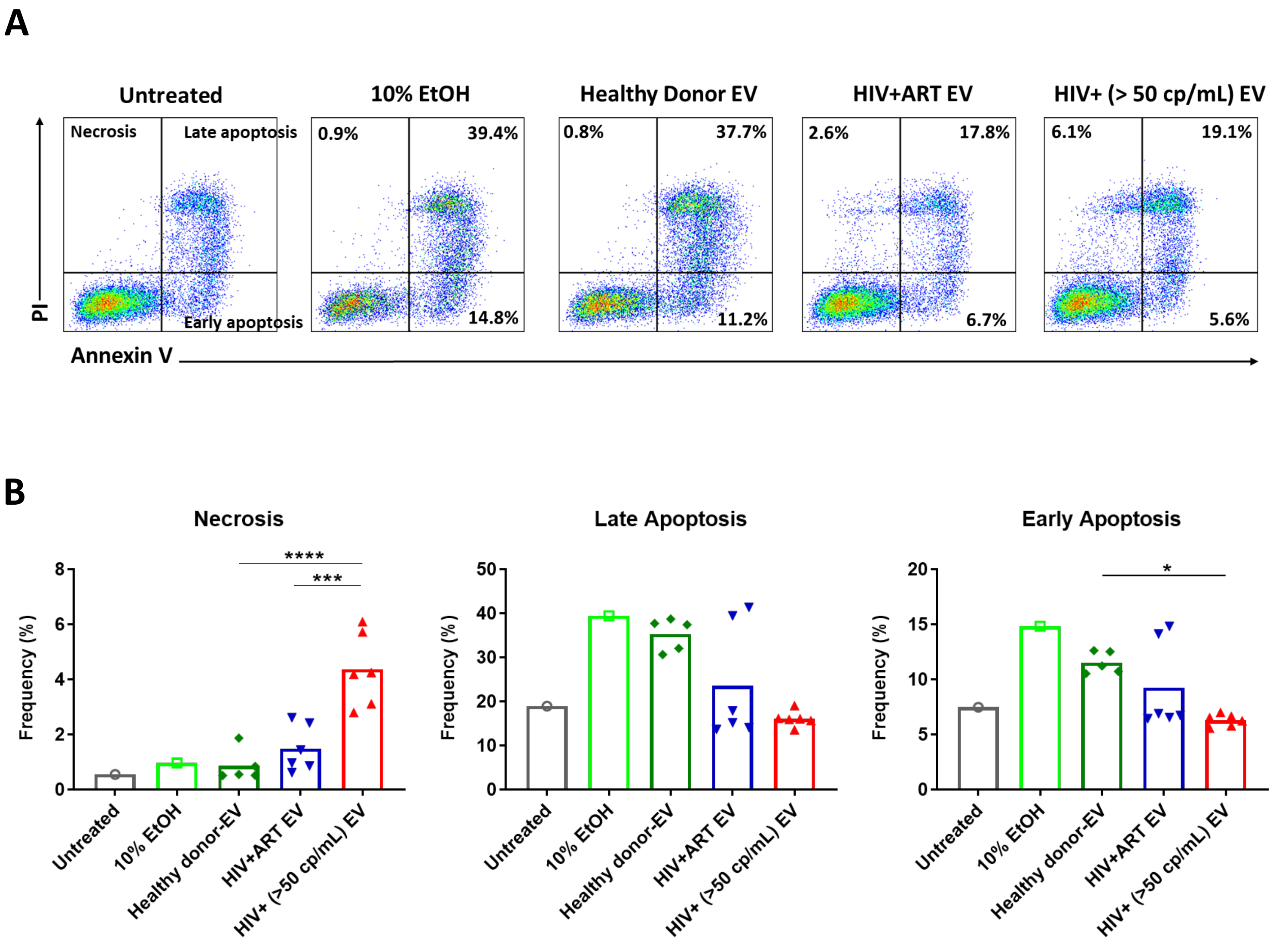

Figure S3: Effect of EVs derived from HIV-infected individuals with a high viral load on HUVECs death. (a) Representative gating by flow cytometry for HUVECs untreated or treated with 10% ethanol (EtOH), EVs from HIV uninfected individuals, EVs from HIV-infected individuals on suppressive antiretroviral therapy (ART), and EVs from HIV-infected individuals with high viral. (b) Comparisons were evaluated using Kruskal-Wallis test and Dunn’s multiple comparison post hoc test. Statistical significance is indicated as *P <0.05, ***P <0.001, ****P <0.0001.
